# Controversies in Breastfeeding

**DOI:** 10.3389/fped.2018.00278

**Published:** 2018-11-01

**Authors:** Riccardo Davanzo

**Affiliations:** ^1^Division of Pediatrics and Neonatology, Department of Mother and Child Health, Ospedale Madonna delle Grazie, Matera, Italy; ^2^Task Force on Breastfeeding, Ministry of Health, Rome, Italy

**Keywords:** breastfeeding, contraindication, maternal health conditions, reproductive health conditions, maternal infections, cytomegalovirus, chemical substances

## Abstract

When addressing the compatibility of breastfeeding with certain maternal conditions, we need to differentiate between “contraindication” and “obstacle.” Failure to distinguish between the two confuses new mothers and their families, and engenders misconceptions about breastfeeding advice by health professionals. Health conditions that may simply impede the initiation and duration of breastfeeding are often wrongly referred to as true contraindications to breastfeed, under the assumption that they might harm the health of the mother and/or that of the nursing infant. Here, we discuss several topics, including breast surgery, prolactinoma, concurrent new pregnancy, hormonal contraception, and use of medications and contrast agents, that continue to raise controversy. While most conditions appear to be compatible with breastfeeding, the major determinants of a woman's final choice of whether to nurse her infant or not are the attitude of health professionals and the state of mind of being an informed mother.

## The concept of contraindication

Breastfeeding, because of its strong health-promoting effect on both the mother (1) and the child (2), affords individual, familial, and social benefits that carry significant economic advantages (3–5). International health authorities (6) and national scientific societies (7) recommend breastfeeding as the nutritional norm, unless an informed choice of the mother or a good medical reason exists for preferring formula feeding (8). There are relatively few evidence-based medical reasons for the use of breastmilk substitutes, yet popular perceptions and beliefs, as well as common attitudes among health professionals, have raised unwarranted concern about breastfeeding. Consequently, many mothers do not start and/or do not continue to breastfeed owing to the confusion between proven and presumed acceptable medical reasons for formula feeding.

When approaching the issue of compatibility of breastfeeding, we need to differentiate between “contraindication” and “obstacle.” Failure to distinguish between the two confuses new mothers and their families, and engenders misconceptions among health professionals. All too often, conditions that may simply impede the initiation and the duration of breastfeeding are wrongly taken as true contraindications to breastfeed, under the assumption that they could harm the health of the mother and/or that of the nursing infant. The term contraindication refers to “something (a symptom or condition) that is a medical reason for not doing or using something (a treatment, procedure or activity)”^1^; for example, maternal diabetes is not a contraindication to breastfeeding. In brief, a contraindication is a specific situation in which a drug or a procedure should not be used because it may be harmful for the person. A contraindication can be either relative, when a situation is acceptable and the benefits outweigh the risks, or absolute, when the risks are definitely unacceptable.

Quite different from this is the concept of obstacle: an obstacle is “an object that you have to get around or over: something that blocks your path”^1^. For example, a breastfeeding mother may have to cope with mastitis or a tiny baby that is not competent to feed at the breast. These constitute simple obstacles rather than contraindications, although such obstacles might sometimes be very hard to overcome.

The list of true contraindications to breastfeeding is short, clearly stated, and easily available from authoritative scientific sources (7)^2^ (Table 1); however, health professionals continue to give contradictory advice (9). Here, we review the compatibility of breastfeeding with conditions in which it is often disfavoured or discouraged. The aim is to provide a more balanced perspective on the barriers and challenges to initiating or continuing breastfeeding (Table 2).

**Table 1:** Contraindications to breastfeed.

1. *Mothers should NOT breastfeed*
• Infant galactosemia
• Mother HIV or HTLV positive
• Mother is using an illicit street drug
• Mother has Ebola virus disease
2. *Mothers should temporarily NOT breastfeed*
• Mother with untreated brucellosis
• Mother is taking certain medications
• The mother is undergoing diagnostic imaging with radiopharmaceuticals
• Mother has herpes simplex virus (HSV) lesions present on the breast (Note: Mothers can breastfeed directly from the unaffected breast)
3. *Mothers should temporarily NOT breastfeed, but CAN feed expressed breast milk*
• Mother has untreated, active tuberculosis. The mother may resume breastfeeding when no longer contagious
• Mother has varicella infection at delivery (5 days prior to delivery to the 2 days following delivery).

*Modified from the US National Center for Chronic Disease Prevention and Health Promotion (CDC)^2^*.

**Table 2:** A selected list of controversial contraindications to breastfeed, discussed in the present paper.

Mother health conditions
• Breast augmentation
• Breast reduction
• Previous breast cancer
• Maternal prolactinoma
Reproductive health conditions
• Pregnancy
• Hormonal contraception
Maternal infections
• CMV infection
• HIV infection
• ZIKV infection
Chemical substances in human milk
• Drugs
• Contrast media agents

## Maternal health conditions

### Breast augmentation

Augmentation mammoplasty is a surgical procedure that increases breast size by inserting breast implants under the breast tissue or chest muscles (10). For some women, breast augmentation is a way to enhance self-image and self-confidence. For others, it is part of breast reconstruction after breast cancer surgery or other conditions affecting the breast. Compared to the general population of new mothers, women who had undergone breast augmentation stated they experienced no differences in attempting to breastfeed (11); however, they were noted to have a lower rate of breastfeeding at maternity discharge (79 vs. 89%) (12) and a lower rate of exclusive breastfeeding at 1 month after childbirth (54 vs. 80%) (12).

### Reduction mammoplasty

Reduction mammaplasty, commonly known as breast reduction, is a procedure by which excess breast fat, glandular tissue, and skin are removed to achieve a breast size more in proportion with the woman's body and to alleviate the discomfort associated with excessively large breasts (13). Disproportionately large breasts can cause both physical discomfort (due to breast weight) and emotional distress. Most women choose to undergo reduction mammoplasty simply for cosmetic reasons.

Whatever the reason for undergoing mammoplasty, breastfeeding after breast reduction surgery might be challenging, although certain surgical techniques can help preserve the mother's ability to breastfeed. Studies conducted in Brazil (14, 15) suggest that breast reduction surgery is associated with negative breastfeeding performance: compared to controls, women who underwent reduction mammoplasty interrupted exclusive breastfeeding after a median duration of only 5 days and were less disposed to exclusively breastfeed at 4 months (22 vs. 4%) after childbirth.

A systematic review of 51 observational studies found the impact of breast reduction surgery (via 31 different breast reduction techniques) on breastfeeding success to vary widely (4–75%) (16). An important determinant was preservation of the subareolar parenchyma, i.e., the column of parenchyma from the nipple areola complex to the chest wall. When the subareolar parenchyma was not preserved, only 4% of women could be expected to breastfeed successfully (16).

### Previous breast cancer

Breastfeeding and breast cancer may be linked in two different ways. First, the current literature reports a protective effect of breastfeeding against breast cancer: the relative risk of breast cancer decreases by 4.3% for every 12 months of breastfeeding and by 7.0% for each birth (17). Second, breast cancer survivors may want to become pregnant and possibly to breastfeed. Over the last decade researchers have assessed the risk of stimulation of the mammary gland following lactation, mainly for reactivation of tumorogenesis, and explored this association (18, 19). In their multicenter case-control study, Lambertini et al. (20) compared 333 patients who became pregnant after surviving breast cancer and 874 nonpregnant patients. They found no difference between disease-free survival and overall survival at a follow-up of 7.2 years after pregnancy. In addition, time to pregnancy following breast cancer treatment and breastfeeding was ascertained to be safe.

Summarizing, cosmetic breast surgery and breast cancer surgery do not constitute a contraindication to breastfeed, although the former may be associated with difficulties in starting to breastfeed and with a shorter duration of breastfeeding because of the altered anatomy of the mammary gland and possible body image concerns (21). Mothers who experience the changes to their body during pregnancy as negative may be less likely to plan or initiate breastfeeding because of the perceived impact of feeding upon their appearance (21).

### Prolactinoma

Prolactin (PRL) is a hormone normally released by the anterior pituitary gland after nipple stimulation during a breastfeed (22). Prolactinoma, a pituitary tumor, is one of the most common causes of prolactin excess and results in hypogonadism, infertility, and galactorrhea. As lactation stimulates PRL production, it has been questioned whether breastfeeding might promote the growth of a preexisting prolactinoma.

Opinions on the compatibility of a prolactinoma with breastfeeding differ, as documented by an online survey conducted in the Middle East and North Africa among 468 physicians, 36% of which were endocrinologists (23). Survey results showed that 47% of responders would allow breastfeeding without restrictions, 28% would allow breastfeeding only by patients with microprolactinomas, and 25% would not recommend it at all (23).

Treatment of hyperprolactinemia with dopamine agonists (DAs)(bromocriptine or cabergole) restores fertility in over 90% of cases. Although DAs have a good safety profile during early pregnancy, they are discontinued when a woman becomes pregnant. The risk of prolactinoma enlargement during pregnancy is very low (2–3%) for microprolactinomas but much higher for macroprolactinomas (20–30%) (24). Careful follow-up by magnetic resonance imaging (MRI) and fundoscopic examination of the prolactinoma is advised during pregnancy. If enlargement does occur, reinitiation of DA therapy is advised but it may be delayed as long as breastfeeding is desired. Finally, breastfeeding *per se* has no harmful effect on tumor growth (25).

In their retrospective study on 107 pregnancies of 73 patients with prolactinoma (54 microprolactinoma; 19 macroprolactinoma) (26), Domingue et al. performed MRI prior to pregnancy and at a median follow up of 22 months after delivery or cessation of lactation. MRI at follow-up showed either no tumor (23%) or a decreased adenoma (39%) in the majority of patients. Morevover, the number of pregnancies per woman, breastfeeding and its duration did not influence the remission rate. In conclusion, lactation is compatible with a previous diagnosis of prolactinoma, with no need to limit duration of breastfeeding.

## Reproductive health conditions

### Breastfeeding during pregnancy

Women may be still breastfeeding when they become pregnant again (27). This creates the dilemma of whether to breastfeed or not, as there is millennial cultural and medical pressure to discourage women from breastfeeding during pregnancy. According to Soranus of Ephesus, a famous physician of the Roman Empire, “a woman who nurses the infant either grows prematurely old, having fed one child, or the expenditure for the nourishment of the offspring necessarily makes her own body quite emaciated.” (28) This view of the adverse effects of breastfeeding on women's health deeply influenced not only ancient Roman society but has permeated European culture throughout the following centuries until today.

We can understand that breastfeeding during pregnancy may be viewed as a challenge to maternal well-being. The main medical reasons, however, regard maternal nutrition depletion, spontaneous abortion, reduced fetal growth, preterm delivery, impaired quality or quantity of mother's milk, and reduced growth of the nursing infant. Owing to the scarcity of scientific literature on the possible medical effects of breastfeeding during pregnancy, clear medical guidelines on this topic are lacking. To overcome this gap, the Italian Society of Perinatal Medicine (SIMP) Working Group on Breastfeeding and the Task Force on Breastfeeding of the Italian Ministry of Health have reviewed the available literature on breastfeeding during pregnancy and issued a position statement (29). There is no evidence indicating that breastfeeding during pregnancy raises the risk of miscarriage or preterm delivery or intrauterine growth restriction, particularly in women from developed countries. Both the composition of postpartum breast milk and the growth of the newborn might be affected, chiefly in women from developing countries (29). Moreover, we must underline that the impact of breastfeeding during the third trimester of pregnancy has been overemphasized, as it is no longer expected to be exclusive breastfeeding when the nursed infant is older than 6 months of age and possibly already being weaned (30).

In brief, breastfeeding during pregnancy is compatible during the first two trimesters, and it is sustainable in the third trimester unless maternal nutrition is suboptimal or the risk of premature delivery exists (multiple gestation, intrauterine growth retardation, previous preterm delivery) (29).

### Hormonal contraception

Most women who breastfeed exclusively stop having menstrual periods (lactational amenorrhea) and have a lower potential for ovulation; nevertheless, the risk of becoming pregnant cannot be excluded. Some sort of contraceptive method is needed if the mother does not plan to become pregnant again (31). Barrier methods (diaphragms, condoms, etc.) have no effect on milk production and so can be used as a first choice contraceptive method by the breastfeeding mother. Their efficacy is inferior to hormonal contraceptives, however.

Many women incorrectly believe that breastfeeding in itself precludes taking any form of hormonal contraception, although a distinction must be drawn among the many contraceptive methods available. Combined hormonal contraceptives (CHCs) may adversely affect milk supply, making the outcome of breastfeeding unpredictable. As some mothers can completely dry up, women are advised to use the lowest appropriate estrogen dosage and monitor their milk supply. Progesterone only pills (POP) are preferred as they are less likely to reduce milk supply (32), an adverse effect, that is even less welcome in the breastfeeding mother with a basic low milk production or during the first 2 months postpartum when the milk supply is still calibrated.

A contraceptive method's safety should be determined in the context of relevant conditions, including phase of lactation, increased thrombotic risk during the postpartum period, and woman's lifestyle. In other terms, the safety of a contraceptive method should be weighed along with the benefits of preventing unintended pregnancy as indicated by the medical eligibility criteria (MEC) for contraceptive use (33). Table 3 presents the categories of the United States Medical Eligibility Criteria for Contraceptive Use (US MEC). Table 4 presents the MEC for hormonal methods in breastfeeding postpartum women. In conclusion, both CHCs and POP, although POP is preferable, can be safely used by breastfeeding women after the first 42 days postpartum.

**Table 3:** US MEC Categories-2016.

1. No restriction for the use of the contraceptive method for a woman with that condition
2. Advantages of using the method generally outweigh the theoretical or proven risks
3. Theoretical or proven risks of the method usually outweigh the advantages – not usually recommended unless more appropriate methods are not available or acceptable
4. Unacceptable health risk if the contraceptive method is used by a woman with that condition


**Table 4:** US-MEC (2016) for CHCs and POP in the breastfeeding woman with and without risk factors for venous thromboembolism (VTE).

**Time post-partum**	**CHCs**	**POP**
<21 days postpartum	4	2
21–30 days postpartum		
• With other risk factors for VTE	3	2
• Without other risk factors for VTE	3	2
30–42 days postpartum		
• With other risk factors for VTE	3	1
• Without other risk factors for VTE	2	1
>42 days postpartum	2	1

## Maternal infections

### Cytomegalovirus infection

Between 37 and 93.7% of pregnant women are CMV-IgG seropositive (34) and more than 50% of CMV-IgG positive mothers produce CMV-positive breastmilk (35). CMV can be isolated in milk whey and from milk cells, mainly neutrophils (predominant during the first 30 days of lactation) and macrophages (predominant starting 60 days postpartum onwards) (36). Although human milk contains biological factors (e.g., lactoferrin) that are known to protect against viral infection, inhibition of CMV virulence is only partial and mother-to-child transmission of CMV infection is still possible (37).

Postnatal CMV infection with maternal infected liquids (milk, saliva, urine) rarely causes severe clinical illness in full-term infants (38) and it is usually devoid of relevant late sequelae. Differently, postnatal CMV infection in immunodeficient infants, particularly in moderate-severe preterm infants and/or very low birth weight infants (VLBWIs), was believed until recently to carry significant short- (39) and long-term health consequences (40). In their meta-analysis of studies (41) on mother-to-child transmission of CMV infection among VLBWIs, Lanzieri et al. reported that the risk of transmission is higher with fresh breastmilk (19%; range 11–32%) and lower with frozen (−20°C) breastmilk (13%; range 7–24%). Among the VLBWIs infected with CMV, 4-5% developed a major short-term illness resembling a sepsis like-syndrome (41). Freezing the milk inconsistently reduces CMV infectivity (42, 43), especially when the viral load is high. In contrast, Holder pasteurization (30 min at 62.5°C) completely destroys CMV infectivity in human milk, reducing the transmission rate to almost null (44). Although pasteurized banked human milk from CMV-negative donors may be the least riskiest option of transmission of CMV from the mother to a VLBW infant, the process adversely affects the bioactive properties of human milk. Recently, high-temperature short-time (HTST) pasteurization has been proposed as an alternative method to better preserve some of the biological components of human milk (45, 46).

Regarding the debate over the long-term cognitive consequences of postnatal CMV infection through human milk in preterm infants, a recent large prospective study conducted in the Netherlands involving 356 preterm infants with a gestational age of <32 weeks followed-up until age 6 years reported that neurodevelopmental problems, including hearing loss, are unlikely (47). As pasteurization or freezing reduces the biological and immunological protective value of breastmilk against necrotizing enterocolitis (48), the study concluded that these processes may not be justified (47).

Although options differ (Table 5) (49) on how to feed a VLBWI of a CMV-seropositive mother, the value of routine feeding of human milk to preterm infants outweighs the risks of clinical disease (2). Withholding the use of fresh breastmilk in the nutrition of VLBWIs no longer seems justifiable.

**Table 5:** Options when feeding the VLBWI with CMV positive mother's milk.

**Option**	**Comment**
1. Pasteurization	• Expensive • Effective • Biological pauperization
2. Freezing before use	• Cheap • Limited effectiveness
3. Application of a MTC transmission protocol, which includes: • Weekly CMV urine test until 32 wks PCA • Informed choice of parents • Weekly CMV urine monitoring by polymerase chain reaction • If the infant becomes positive, fresh milk feeding possibly stopped to reduce viral loads (49)	• Cumbersome • Ambiguous • Causing anxiety
4. Use of fresh mother's milk, in any case	• Priority to the prevention of NEC • Favorable risk/benefit ratio

### Human immunodeficiency virus (HIV) infection

Although breastfeeding is one of the most valuable ways to enhance child survival, until recently HIV-positive mothers in developed countries were discouraged to breastfeed, as mother-to-child transmission of HIV infection can occur not only during pregnancy and delivery but also through breastfeeding (50, 51). Accumulating evidence from countries with a high prevalence of HIV infection in the population of pregnant women has shown that giving antiretroviral medicines to mothers and baby can significanlty reduce the risk of HIV transmission through breastfeeding (52). In a meta-analysis of the postnatal-mother-to-child transmission (PMTCT) rate of HIV infection, the overall pooled transmission rates for breastfed infants with mothers on antiretroviral treatment (ART) were 3.5 and 4.2% at 6 and 12 months, respectively (53). A substantial reduction in postnatal HIV transmission risk under maternal ART was noted (PMTCT rate 1.1% at 6 months and 2.9% at 12 months) (53). The risk of PMTCT was noted to increase once ART was stopped—usually at 6 months—supporting current recommendations that all women diagnosed as HIV-infected should receive immediate and lifelong ART.

A recent randomized trial involving 14 sites in Sub-Saharan Africa and India compared the efficacy of prolonged infant antiretroviral prophylaxis with nevirapine vs. maternal ART for the prevention of mother-to-child transmission (MTCT) throughout the breastfeeding period (54). Maternal ART or infant nevirapine (iNVP) prophylaxis was safely continued until 18 months after delivery or breastfeeding cessation. The MTCT rate at 24 months was very low in both study arms (ART 0.57%; iNVP 0.58%).

In view of this evidence, in 2016 the World Health Organization (WHO) released updated guidelines on HIV and infant feeding (55). Pregnant women who regularly receive ART before delivery are expected to have a low enough number of HIV copies in their blood that poses a negligible risk of transmission of the virus during labor and delivery, so that they may have a normal vaginal birth and might also eventually breastfeed. According to WHO recommendations, infants born to HIV-positive mothers can be exclusively breastfed for the first 6 months of life, with the introduction of appropriate complementary foods thereafter; they can continue to be breastfed for up to 24 months or longer while the mothers are being fully supported for ART adherence (55).

WHO guidelines refer to limited recourse countries where breastfeeding is the cultural norm and formula feeding is stigmatized in the local community. Though HIV-seropositive status constitutes a potential contraindication to breastfeed, there are good medical as well as social reasons to encourage HIV-positive mothers to breastfeed. Moreover, also in industrialized countries an HIV-positive mother can be supported to breastfeed if she adheres to ART and has a low plasma viral load. When these criteria are met, the postnatal transmission rate at 12 months after delivery for the breastfed infant might be around 3% and even lower if ART is maintained for between 6 and 12 months (52, 54).

Although breastfeeding is still a documented risk factor for MTCT of HIV (56), the British HIV Association (57) and the American Academy of Pediatrics (58) recommend that mothers who choose this option should practice exclusive breastfeeding for no more than 6 months while undergoing regular monitoring of maternal ART compliance, maternal viral load, and infant HIV status. In conclusion, encouraging breastfeeding while the HIV-positive mother is on ART is feasible and may become common practice in the near future also in industrialized countries.

### ZIKV infection

The Zika virus (ZIKV) is a mosquito-borne (*Aedes*) RNA flavivirus that causes a “dengue-like syndrome” as a usual clinical manifestation (59). ZIKV has the potential to spread to areas where the *Aedes* mosquito vector is present, including Southern Europe. ZIKV represents an emerging health threat, particularly due to associated neurological disease in newborns and adults. Consequences of vertical infection include microcephaly with brain and eye abnormalities, and consequences of adult infection include Guillain-Barré syndrome and meningoencephalitis. The route of transmission of ZIKV is multiple (60): sexual, via blood transfusion, intrauterine, perinatal (still unknown spectrum of clinical features), postnatal due to mosquito sting and possibly to breastfeeding.

Following the identification of ZIKV in breastmilk (61, 62), its role as a potential route of transmission has been questioned. Although paucisymptomatic ZIKV infection has been described anecdotally in breastfed infants (63, 64), no proven MTCT of infection via breastmilk has been confirmed. In conclusion, as the benefits of breastfeeding outweigh the risk of possible ZIKV transmission via breastmilk, the WHO (65) and the United States Centers for Disease Control and Prevention (CDC)^3^ consider maternal ZIKV infection compatible with breastfeeding.

## Chemical substances in human milk

### Drugs

Pharmaceutical companies rarely give complete information about the appearance of a drug in breastmilk following assumption by the mother and about the possible side effects for the nursing infant. Drug companies do not provide more detailed information because they choose not to study the problem. Consumers are expected to read the lengthy precautions on the package insert that indicate that the drug should not taken during pregnancy and lactation (66). According to the information in the European Summaries of Product Characteristics (SmPCs), the use of 90% of medicines is restricted during both pregnancy and breastfeeding, despite a lack of information to support such indications (67). This defensive position is taken to avoid possible litigation.

It comes as no surprise, therefore, that the use of medications by a nursing mother may be a valid reason for breastfeeding cessation, albeit arguably stemming from an excessively cautious approach. Although almost all drugs pass into breastmilk, the known adverse effects in infants are relatively few. Most adverse effects are reported as being merely associated, without having a certain causal relationship.

Moreover, many side effects are described in the first 2 months of life (68) when breastfeeding is still exclusive or nearly exclusive, limiting the relevance of the passage of a drug into the mother's milk at an older age when semisolids and solids are added to the diet, thus reducing the possible drug burden on the infant.

When documented clinical data on side effects in the infant are unavailable, pharmacokinetic parameters provide the theoretical basis on which the lactation risk is initially assessed (69). From a pharmacological standpoint, we can appreciate that the minimum infant intake of a maternal drug through breastmilk can be expected when its half-life is short, maternal plasma protein binding is high (allowing less unbound drug to pass into the milk), the biochemical characteristics of the drug lead to a low milk-to-plasma ratio, and absorption by the infant gut is slow, resulting in poor bioavailability.

Most drug transfer from the mother's plasma into breastmilk, although rarely exceeding the relative infant dose (RID) of 1 percent of the mother's dose, much less than the upper limit of safety, that is 10% of the maternal dose (69). RID is calculated by dividing the infant dose via milk in mg/kg/day by the maternal dose in mg/kg/day, assuming a mother's body weight of 70 kg.

When giving advice or counseling on the use of medications during breastfeeding, health professionals should use the most authoritative information source that provide pharmacological and toxicological information. LactMed^4^ appears to be the most reliable source for quality of citations. After a methodologically appropriate assessment of the safety of a medication in a breastfeeding mother, there is no conflict of interest between the right of the mother to self-treat and the health of her breastfed infant in most cases (70). Nevertheless, assessing the safety profile of a drug in breastfeeding women requires investment by health professional in terms of time, specific scientific knowledge, and empathic approach.

Compared to their older colleagues, few young pediatricians feel that mothers can successfully breastfeed (70% in 1995 vs. 57% in 2014) and that the benefits of breastfeeding outweigh the difficulties (70% in 1995; 50% in 2014) (71). Ultimately, a disfavorable attitude of pediatricians toward the promotion of breastfeeding may hinder the development of well-balanced professional counseling on medication use during breastfeeding. Furthermore, health professionals should also appreciate that some mothers may have a limited willingness to invest in their breastfeeding experience and will ultimately make an informed choice for bottle feeding.

### Contrast agents

Contrary to mistaken beliefs that breastmilk is altered by radiation, medical imaging including computed tomography (CT) does not affect the quality of breastmilk or the health of the breastfed infant. Where some uncertainty does, in fact, exist regards the possible health consequences of the passage into breastmilk of contrast agents for imaging studies (CT or MRI). Breastfeeding mothers who require intravascular iodinated or gadolinium-based contrast for an imaging procedure are usually advised to interrupt breastfeeding for 24 to 48 h after exposure to the contrast agent. Because of ethical considerations, no controlled trials have directly examined the safety of breastfeeding after imaging with radiocontrast agents to date.

According to the American College of Radiology (ACR), a negligible dose of contrast agent administered intravenously to the mother is absorbed by the gastrointestinal tract of the breastfed infant: <0.01% for iodinated X-ray contrast agents and <0.0004% for gadolinium-based contrast agents (72). Although the final dose of contrast medium absorbed by a breastfeeding infant whose mother receives an intravenous agent is not expected to pose significant toxic or allergic harm to the breastfed infant, the ACR still recommends the option of abstaining from breastfeeding for a period of 12–24 h if this is the preference of an informed mother (72).

An Italian joint working group on the administration of contrast agents to breastfeeding women has come to different conclusions (73), considering the majority of contrast agents safe, except for gadopentetate dimeglumine, gadodiamide, and gadoversetamide. Only these three contrast agents should be precautionally avoided in breastfeeding women due to the high risk of nephrogenic systemic fibrosis (73), although its occurrence has not been reported in infants or young children (74). After MRI or CT examination with a contrast agent: (1) breastfeeding should be temporarily discontinued, the breastmilk expressed and discarded in a limited number of cases; (2) there are no evidence-based reasons for the routine suspension of breastfeeding.

## Conclusion

The present review provides useful information for developing sound advice for the breastfeeding mother in the context of controversial conditions that are uncritically accepted as true contraindications to breastfeeding. Most conditions appear to be safe and compatible with breastfeeding. The major determinants in the final choice are the attitude of the health professionals consulted and the state of mind of the informed mother.

## Author contributions

RD conceived the project and made a substantial, direct and intellectual contribution to the work, and approved it for publication.

### Conflict of interest statement

The author declares that the research was conducted in the absence of any commercial or financial relationships that could be construed as a potential conflict of interest.

## Footnotes

^1^https://www.merriam-webster.com/

^2^https://www.cdc.gov/breastfeeding/breastfeeding-special-circumstances/contraindications-to-breastfeeding.html (Accessed June 30, 2018).

^3^https://www.cdc.gov/pregnancy/zika/testing-follow-up/zika-in-infants-children.html

^4^https://toxnet.nlm.nih.gov/newtoxnet/lactmed.htm
